# Pediatric subset of primary immunodeficiency patients treated with SCIG: post hoc analysis of SHIFT and IBIS pooled data

**DOI:** 10.1186/s13223-020-00478-2

**Published:** 2020-09-09

**Authors:** Viviana Moschese, Clementina Canessa, Antonino Trizzino, Baldassarre Martire, Giorgio Maria Boggia, Simona Graziani, the SHIFT and IBIS Study Groups

**Affiliations:** 1 Pediatric Immunopathology and Allergology Unit, University of Rome Tor Vergata, Policlinico Tor Vergata, Viale Oxford, 81, 00133 Rome, Italy; 2 Pediatric Immunology Meyer Children’s Hospital University of Florence, Viale Pieraccini 24, 50139 Florence, Italy; 3 Department of Pediatric Hematology and Oncology, ARNAS Civico Di Cristina and Benfratelli Hospital, Palermo, Italy; 4 Pediatric Unit, “Mons. Dimiccoli” Hospital, Viale Ippocrate, 70051 Barletta, Italy; 5 Medical Affairs, CSL Behring, Milan, Italy

**Keywords:** Pediatric patients, Primary immunodeficiency, SHIFT study, IBIS study, Immunoglobulin, Infection rate

## Abstract

**Background:**

Primary immunodeficiencies (PID) constitute a heterogeneous group of more than 350 monogenetic diseases. PID patients with antibody impairment require lifelong administration of immunoglobulin G replacement therapy, administered either intravenously (IVIG) or subcutaneously (SCIG). Although the effectiveness of weekly and biweekly (every other week) SCIG administration has been shown in several trials, data on the viability of these two regimens in pediatric PID patients are sparse.

**Methods:**

Data on the pediatric subsets of PID patients enrolled in SHIFT (weekly) and IBIS (biweekly) studies were pooled and analyzed to indirectly compare two different 20%-concentrated SCIG (Hizentra^®^) regimens. The primary endpoints were to evaluate trough IgG levels and cumulative monthly doses; the secondary endpoint was to analyze incidence of infections.

**Results:**

Fifteen and 13 children from the SHIFT and IBIS studies were included, respectively. Cumulative 20%-concentrated SCIG monthly dose was slight lower for the biweekly regimen (Δ = − 2.04, 90% CI − 8.3 to 4.23). However, the trough IgG levels were similar between the two groups (Δ = 0.28, 90% CI − 0.51 to 1.07) and constantly above the threshold of 5 g/L. After adjusting for potential confounders, the annualized rate of infections was similar between SHIFT and IBIS patients (incidence rate ratio = 1.09, 90% CI 0.72–1.67); only 1 serious bacterial infection was experienced by a patient in the IBIS group.

**Conclusion:**

In pediatric PID patients, weekly and biweekly Hizentra^®^ administrations appeared equally effective treatment options.

## Introduction

Primary immunodeficiencies (PIDs) include several genetic and immune dysregulation disorders that affect various components of the innate and adaptive immune systems. Predominant antibody deficiencies (PAD) are the most common PID defects and are characterized by an impairment of B-cell development and function. Most patients have hypogammaglobulinemia and suffer from recurrent bacterial infections, mainly affecting respiratory and gastrointestinal tracts.

Immunoglobulin G (IgG) replacement therapy is considered an “essential” medication to be administered lifelong [1] in order to achieve the highest possible protection against infections, including serious bacterial infections (SBIs), i.e., bacteremia/sepsis, bacterial meningitis, osteomyelitis/septic arthritis, bacterial pneumonia, and visceral abscess [2]. The Food and Drug Administration (FDA) set the SBI rate at 1.0 per person-year, and treatments with values below this threshold are considered effective [2]. Although a trough serum IgG level of 5 g/L (or higher in some cases) has been historically considered protective against infections [3], a “biological trough level”, meaning the serum IgG level that permits the best clinical status in each individual patient, has been proposed [4].

Currently, two administration routes with different pharmacokinetic properties are licensed: intravenous Ig (IVIG) and subcutaneous Ig (SCIG).

Although they are equally effective [5], each delivery mode has distinct characteristics. While IVIG is preferable when high amounts and prompt correction of IgG are required, SCIG therapy can provide more stable and sustained levels of IgG [6], potentially reducing or avoiding wear-off effects [7]. Moreover, SCIG can be self-administered at home as a cost-effective treatment option [8] and does not require venous access. With regard to safety, SCIG is associated with fewer systemic adverse events and more injection site reactions than IVIG [9].

PID patients typically have a lower quality of life than healthy individuals, and children report more discomfort than parents [10]. In the pediatric population, SCIG showed several advantages over IVIG, including better health and improved school/social functioning; SCIG also reduced parental emotional distress and personal time limitations and exerted fewer limitations on family activities [11].

Hizentra^®^ (CSL Behring, King of Prussia, PA, USA) is a liquid 20%-concentrated SCIG preparation that has been approved as a replacement therapy for PAD. Three pivotal studies [12–14] and their extensions [9] proved its effectiveness and safety when administered weekly.

A pharmacokinetic (PK) and modeling simulation [15] and a subsequent analysis [16] suggested that protective serum IgG trough levels may be reached by administering SCIG through a wide range of intervals, from daily to biweekly (every other week), provided that the total monthly dose is maintained. Later, a retrospective [17] and, more recently, a prospective study including pediatric subjects showed its effectiveness when administered biweekly [18, 19].

The aim of this analysis was to compare the clinical and laboratory features of different Hizentra^®^ dosing intervals in children enrolled in two Italian non-interventional trials.

## Materials and methods

Prospective data from pediatric PID patients (i.e., < 18 years of age) requiring immunoglobulin replacement therapy (IGRT) enrolled in SHIFT [20] and IBIS [18] studies (CSL Behring protocols IgPro20_5001 and IgPro20_5002 respectively) were pooled in a unique database and analyzed. Please refer to the SHIFT [20] and IBIS [18] studies for the complete materials and methods information. Of note, we conducted our post hoc analysis based on sample size justification, rather than calculation, by referring on precedent IBIS and SHIFT observational studies, where sample size calculation was based, as reported, on feasibility. In our paper, by pooling data from all < 18 years old patients from IBIS and SHIFT studies, the pediatric subset has been preserved. In those studies, 2 different regimens of Hizentra^®^ were used: weekly (Q1W) in the SHIFT study and biweekly (Q2W) in the IBIS study.

The objective of this study was to indirectly compare biweekly and weekly administrations of 20%-concentrated SCIG in pediatric patients. The primary endpoints were to evaluate trough IgG levels and cumulative monthly doses; the secondary endpoint was to analyze the incidence of infections during the follow-up period.

For SHIFT patients, since the SCIG dosages at the 3- and 6-month follow-up visits were reported as summary statistics, monthly dosage was retrieved from the screening visit. However, we assumed that during the study, variations in the dosage were negligible because the data in the three visits were similar.

For IBIS patients, the monthly dosage was calculated starting from the mean dose per infusion and the infusion frequency (number of days between two consecutive infusions).

Annualized rates of all types of infections and serious bacterial infections (SBIs), as defined by international guidelines [2], have been considered.

Patients were classified according to their body mass index (BMI), following the growth curves of Cacciari et al. [21], and assuming the 3rd percentile as a proxy of the 5th percentile. The results from the analysis of correlation among the BMI, IgG dosage, and IgG serum levels in the whole SHIFT-IBIS population have been presented elsewhere [22].

Categorical variables were expressed as count and percentages. Continuous variables were summarized using mean ± standard deviation (SD) (median; interquartile range [IQR]), and number of infections and SBIs were expressed as rates, i.e. number of events per person-year (PY). Differences between baseline characteristics of SHIFT and IBIS groups were tested using Chi squared test for categorical variables (Fisher exact test was used in case of < 5 observations in at least one cell) and Wilcoxon test for continuous variables.

Differences in primary and secondary outcomes were estimated using generalized linear regression including age, sex, weight status, baseline serum IgG trough levels (before Hizentra^®^ use), and Hizentra^®^ administration frequency as covariates. Furthermore, only for the infection comparison, we adjusted the relative effect of different administration frequency also for the number of infection at the first enrollment visit. Due to the post hoc nature of the study, for all comparisons we reported the mean relative effect (absolute difference [Δ], risk ratio [RR], or incidence rate ratio [IRR]) and the 90% confidence interval (CI). Analysis and the relevant graphs were created using the statistical software R.

## Results

### Patients’ characteristics

Twenty-eight pediatric patients were present in the pooled database. Fifteen came from the SHIFT study, where a weekly Hizentra^®^ (Q1W) posology was adopted, and 13 came from the IBIS study, where a biweekly (Q2W) regimen was followed. As shown in Table 1, Q1W and Q2W groups had similar demographic data and trough IgG serum levels (before starting treatment with Hizentra^®^).

**Table 1:** Demographic and PID features of 28 children receiving weekly (Q1W) or biweekly (Q2W) Hizentra^®^ treatment

	SHIFT, Q1W (N = 15)	IBIS, Q2W (N = 13)	Difference (90% CI)^a^	Overall (SHIFT-IBIS) (N = 28)
Gender (male)	11 (74.3%)	10 (76.9%)	RR = 1.05 (0.73–1.51)	21 (75.0%)
Age (years)	11.9 ± 4.6 (13; 11–15)	11.5 ± 4.1 (13; 9–14)	Δ = − 0.33 (− 2.99 to 2.33)	11.7 ± 4.3 (13; 10–15)
Height (cm)	148.4 ± 27.8 (157; 138–167)	148.5 ± 24.7 (159; 138–165)	Δ = 0.03 (− 15.97 to 16.02)	148.4 ± 25.9 (158; 136–166)
Weight (kg)	50.3 ± 22.6 (55; 33–63)	47.4 ± 19.8 (47; 34–61)	Δ = − 2.85 (− 15.77 to 10.06)	48.9 ± 21 (49; 33–62)
BMI (kg/m^2^)	21.4 ± 4.7 (23; 17–24)	20.5 ± 4.9 (19; 18–24)	Δ = − 0.82 (− 3.75 to 2.11)	21 ± 4.7 (20; 17–24)
Serum IgG trough levels before Hizentra^®^ use (g/L)	8.2 ± 1.1 (8.3; 7–9)	8.6 ± 2.6 (7.7; 7–10)	Δ = 0.40 (− 1.29 to 2.08)	8.3 ± 1.6 (8.1; 7–9)

Data are reported as the mean ± standard deviation (median; interquartile range) except for the male gender, which is reported as the number of subjects (percentage)

*BMI* body mass index, *CI* confidence interval, *Δ* absolute difference, *Q1W* weekly administration, *Q2W* biweekly administration, *RR* risk ratio

^a^Minimum p-value 0.645

Overall, 21/28 patients were male (75%), with a mean ± standard deviation (SD) age of 11.7 ± 4.3 years. The mean ± SD trough IgG level before starting the Q1W or Q2W administration of Hizentra^®^ in the pooled SHIFT-IBIS pediatric population was 8.3 ± 1.6 g/L; values of Q1W and Q2W were very similar (Δ = 0.40 g/L, 90% CI 1.29–2.08).

**Fig. 1:**
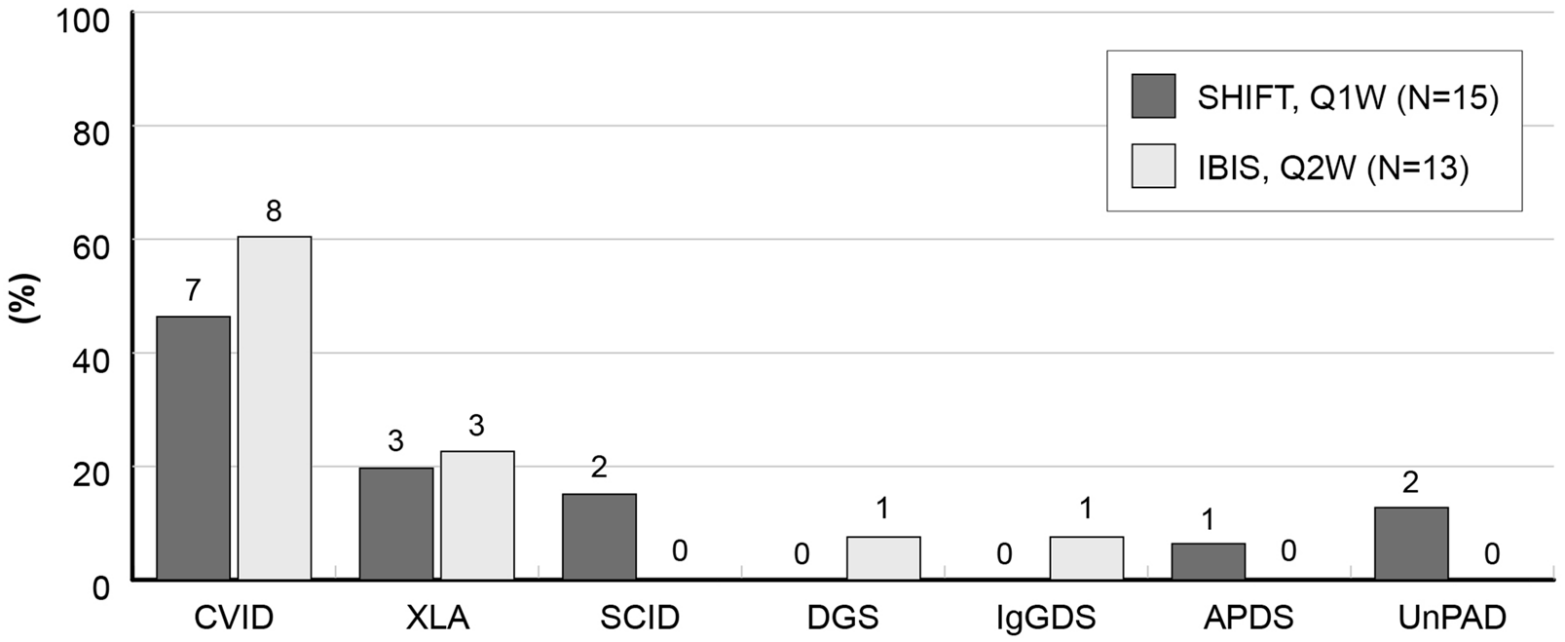
Distribution of primary immunodeficiency conditions. *APDS* activated PI3K-delta syndrome, *CVID* common variable immunodeficiency, *DGS* DiGeorge syndrome, *IgGSD* IgG subclass deficiency, *SCID* severe combined immunodeficiency, *UnPAD* unclassified primary antibody deficiency, *XLA* X-linked agammaglobulinemia

Most prevalent forms of PID were common variable immunodeficiency (CVID), X-linked agammaglobulinemia (XLA) and severe combined immunodeficiency (SCID), all together accounting for 80% of subjects in both groups (Fig. 1).

**Fig. 2:**
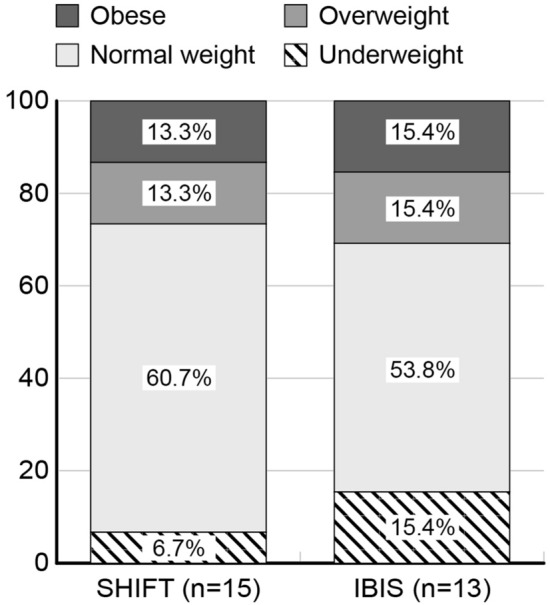
Weight categories of 28 children from the SHIFT and IBIS groups. *Q1W* weekly administration, *Q2W* biweekly administration

The two groups had similar growth parameters. According to BMI, PID children were categorized as underweight if their BMI was below the 3rd percentile, normal weight if their BMI ranged from the 5th–85th percentile, overweight if their BMI ranged from the 85th–97th percentile, and obese if their BMI was > 97th percentile. As shown in Fig. 2, more than 50% of patients in both groups had a normal weight (66.7% in SHIFT and 53.8% in IBIS). Weight distribution seemed balanced between SHIFT and IBIS patients (χ^2^ test, p = 0.8676), excluding a slight lower prevalence of underweighted children in the Q1W group than in the Q2W group (6.7% in SHIFT and 15.4% in IBIS).

### Q1W and Q2W Hizentra^®^ parameters

In Table 2, the Hizentra^®^ infusion parameters with a Q1W and Q2W dosage regimen are reported.

**Table 2:** Characteristics of Hizentra^®^ administration with Q1W and Q2W dosage regimens

	Q1W (N = 15)	Q2W (N = 13)	Difference (90% CI)
Dosage/infusion (g)	4.3 ± 1.2 (4; 3.5–6)	6.7 ± 2.8 (8; 4.5–8)	2.18 (0.88–3.48)
Dosing interval (days)	7.8 ± 2.3 (7; 7–7)	14.9 ± 0.6 (15; 15–15)	6.73 (5.40–8.07)
Monthly dose (g)	18.0 ± 8.8 (16; 12–24)	13.7 ± 5.8 (16; 10–17)	− 2.04 (− 8.30 to 4.23)
Number of infusion sites in parallel	1.7 ± 0.5 (2; 1–2)	1.9 ± 0.3 (2; 2–2)	0.38 (− 0.02 to 0.79)
Infusion length (h)	n.a.	1.6 ± 0.5 (1.5; 1.2–2)	n.a.
Cumulative infusion flow (mL/h)	8.5^a^	24.4 ± 12.2 (23; 18–26)	n.a.
Pump (n.)	n.a.	1.1 ± 0.3 (1; 1–1)	n.a.
Serum IgG trough levels with Hizentra^®^ (g/L)	8.4 ± 1.4 (8.1; 7.2–9.6)	8.5 ± 1.8 (8.4; 7.2–9.8)	0.28 (− 0.51–1.07)

Data are reported as the mean ± standard deviation (median; interquartile range)

*n.a.* non available, *Q1W* weekly administration, i.e., data from the SHIFT study, *Q2W* biweekly administration, i.e., data from the IBIS study

^a^Data available for only 1 subject who was administered therapy at 1 infusion site

**Fig. 3:**
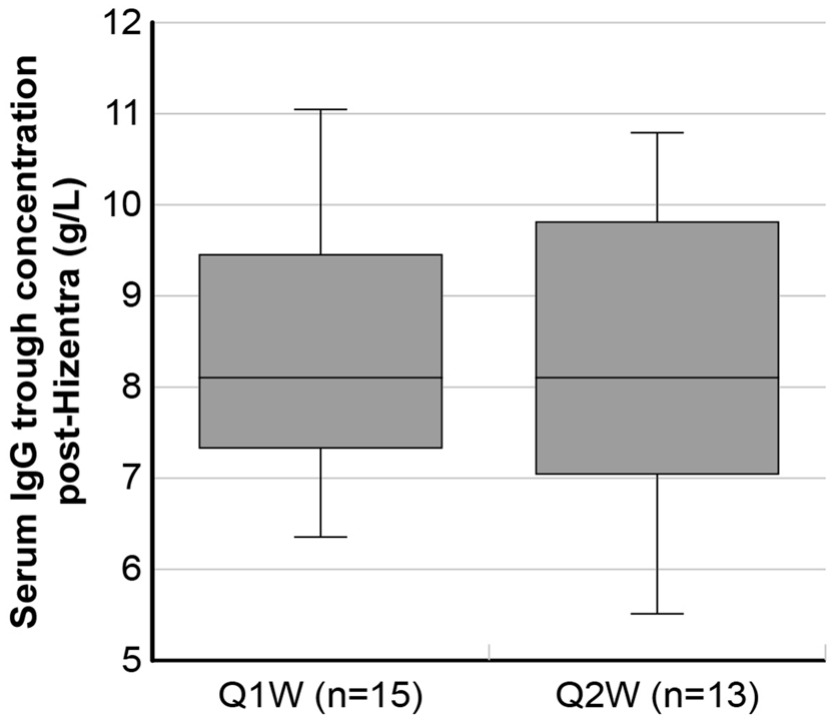
Box plot of serum IgG trough concentrations (g/L) and distributions in the Q1W and Q2W dosage regimen groups. The bold lines represent the median values, the boxes indicate the interquartile range, and the whiskers represent the minimum and maximum values. *Q1W* weekly administration, i.e., data from the SHIFT study, *Q2W* biweekly administration, i.e., data from the IBIS study

Since the dosing interval differed between the Q1W and Q2W groups, a different dosage per infusion was expected (Δ = 2.18, 90% CI 0.88–3.48); the mean ± SD cumulative monthly IgG dose was 18.0 ± 8.8 g and 13.7 ± 5.8 g in Q1W and Q2W, respectively, whilst the number of sites were comparable between the two groups (mean ± SD of 1.7 ± 0.5 and 1.9 ± 0.3, respectively). After adjusting for baseline characteristics, the mean difference in the monthly cumulative dose was − 2.04 g (90% CI − 8.30 to 4.23) and in the number of infusion sites was 0.38 (90% CI − 0.02 to 0.79). All patients had a serum IgG trough level above the threshold of 5 g/L, reported to be protective against most infections. In fact, the mean ± SD serum IgG trough concentrations during Hizentra^®^ treatment were 8.4 ± 1.4 g/L (median = 8.1; IQR = 7.2–9.6) and 8.5 ± 1.8 g/L (median = 8.4; IQR = 7.1–9.8) in the Q1W and Q2W subsets, respectively. The adjusted mean difference was 0.28 g/L (90% CI − 0.51 to 1.07), thus the frequency of administration did not seem to affect the ability to adequately maintain that threshold. In addition, the distribution of serum trough IgG levels was similar between the groups (Fig. 3).

### Infections

The percentages (number) of patients who had at least one infection of any type were 54.5% (6/11) and 76.9% (10/13) in the Q1W and QW2 subsets, respectively (RR = 1.53, 90% CI 0.91–2.59). After adjusting for baseline characteristics, number of ongoing infections at the time of enrollment and follow-up duration, the incidence rate of infection was comparable in the two groups (IRR = 1.09, 90% CI 0.72–1.67) (Table 3).

**Table 3:** Infections in the Q1W and Q2W cohorts

	Q1W (N = 11)^a^	Q2W (N = 13)^b^	Difference (90% CI)
Number of patients with at least 1 infection (%)	6 (54.5%)	10 (76.9%)	RR = 1.53 (0.91–2.59)
Total number of non-SBI (annualized rate)	8 (1.52^c^)	35 (2.68)	IRR = 1.09 (0.72–1.67)
Number of patients treated with antibiotic therapy due to infection	n.a.	8 (61.5%)	n.a.
Days of antibiotic exposure per patient^d^	n.a.	18.9 ± 13.5 (18; 9.25–24.25)	n.a.

The annual rate was calculated as described in the Methods section. Data on antibiotic therapy due to infections were not available in the SHIFT study. Days on antibiotic therapy are reported as the mean ± SD (median; interquartile range)

*IRR* incidence rate ratio, *RR* risk ratio, *SBI* serious bacterial infection

^a^In the Q1W group, 4 patients were not considered, as data on their infections were not available

^b^In Q2W, all patients were considered, but as one patient switched to weekly SCIG therapy at 261 days after enrollment, the relevant infections were considered until only that date and not for 365 days

^c^Annualized rate is calculated in patients with available follow-up lengths (n = 4)

^d^Time of antibiotic exposure per patient was calculated among only those (n = 8) who received antibiotics

**Fig. 4:**
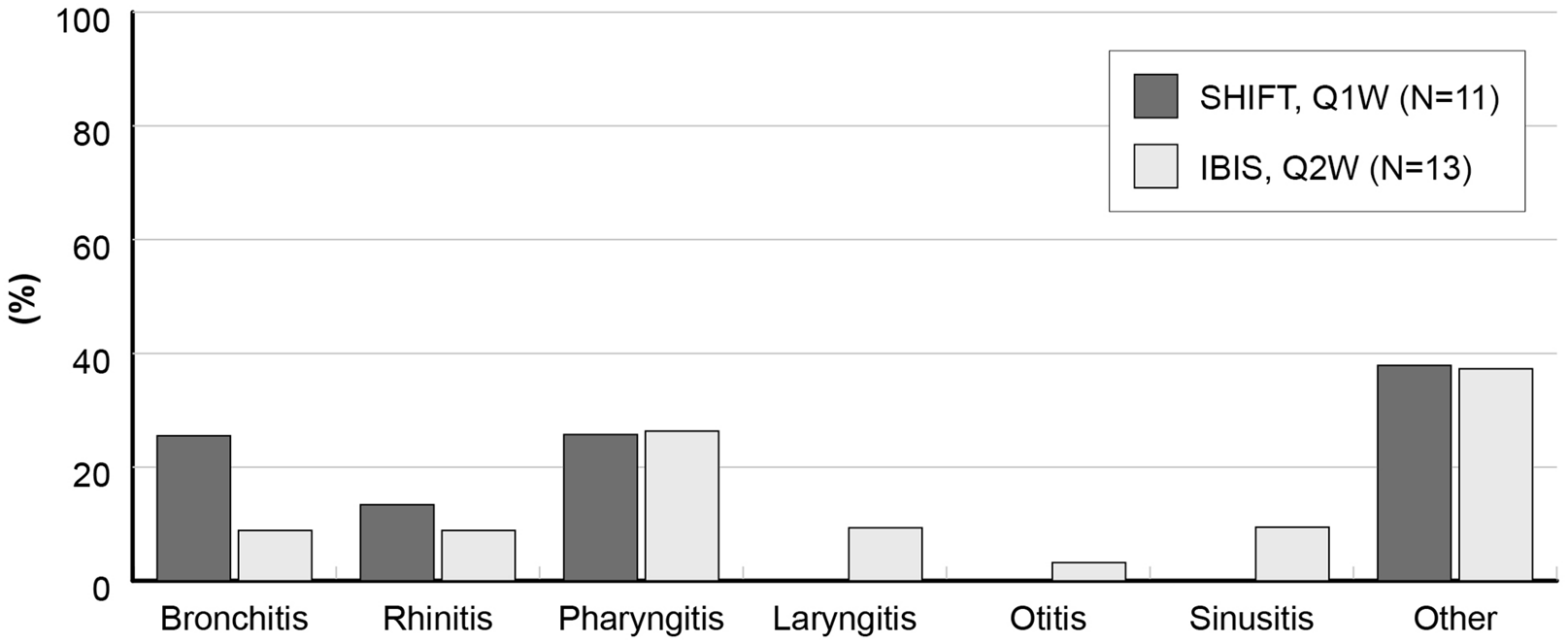
Type and distribution of 8 non-SBI in 6/11 Q1W patients and 35 non-SBI in 10/13 Q2W patients. *Q1W* weekly administration, i.e., data from the SHIFT study, *Q2W* biweekly administration, i.e., data from the IBIS study

In particular, only one SBI was reported in a CVID patient on a Q2W dose regimen, leading to a SBI rate of 0.08 per patient-year, but no SBIs were detected in the Q1W group. Among non-SBI, the most frequent events were bronchitis, rhinitis, and pharyngitis (62.5% in Q1W group and 42.9% in the Q2W group) (Fig. 4). Specific age stratification analysis showed lower infection rate in patients between 10 and 15 years of age (IRR = 0.34, 90% CI 0.21–0.57).

No antibiotic treatment comparisons could be made since data were available for only the Q2W cohort. However, among the IBIS children who received antibiotics (8/13; 61%), the mean ± SD time of antibiotic exposure was 18.9 ± 13.5 days (Table 3).

## Discussion

Bioequivalence between weekly and biweekly SCIG, provided that the monthly IgG dosage was constant, was demonstrated by pharmacometric modeling and simulation [15]. Further simulations demonstrated that AUC, C_max_, and C_min_ were similar for daily and biweekly administrations of SCIG [16].

The first retrospective clinical study with biweekly SCIG administration described 20 PID patients in whom a protective and sustained serum IgG trough level was reached, with a SBI per patient-year rate of 0.036 [17].

While data on weekly Hizentra^®^ administration have been collected in 7 trials for a cumulative period of 250.9 patient-years [13], biweekly SCIG administration in real life has never been studied prospectively before the IBIS study.

The IBIS study [18] used for this analysis was to our knowledge the first 1-year-long prospective observation clinical study to describe IgG levels and clinical parameters in PID patients treated with biweekly Hizentra^®^ (mean trough IgG level of 8.55 ± 1.76 g/L and mean annual rate of SBI of 0.063 ± 0.246).

This post hoc analysis describes 28 PID pediatric subjects treated with Hizentra^®^ in 2 different Italian studies, with an overall exposure of more than 20 patient-years. In comparison, the total number of pediatric patients enrolled in three pivotal phase 3 trials on Hizentra^®^ was 44 [9]. The most prevalent form of PID was CVID, accounting for > 50% of patients, followed by XLA, which explains the slightly higher proportion of males.

A slight reduction in the monthly dose of Hizentra^®^ was observed in the biweekly group with respect to the weekly group (− 2.04 g, 90% CI − 8.30 to 4.23, Table 2); a similar reduction was observed in the IBIS study [18]. However, the mean trough IgG levels among patients treated with weekly and biweekly Hizentra^®^ were absolutely comparable (Δ = 0.28 g, 90% CI − 0.51 to 1.07, Table 2) and above the level considered protective against infections (5 g/L).

In this post hoc analysis, data on infections were available for 11/15 patients from the SHIFT study [20], while an exact observation period was available for 4 patients. Therefore, the annualized infection rate was calculated for 4 SHIFT patients and 12 IBIS patients (another subject had only partial data). Differences in the prevalence of patients with at least one infection (6/11 and 10/13 for Q1W and Q2W groups, respectively, Table 3) were probably due to the shorter follow-up in the Q1W cohort than in the Q2W cohort (mean ± SD days of follow-up were 179.9 ± 9.4 and 366.8 ± 44.5, respectively). In fact, after adjusting for possible confounders, annualized infection rates were similar (IRR = 1.09, 90% CI 0.72 to 1.67). Only one SBI was observed in our pooled database from a subject in the IBIS population, corresponding to a rate equal to 0.08 cases per patient-year; there were not SBI reported in patients receiving weekly Hizentra^®^. These values were far below the threshold of 1.0 SBI per person-year set by the FDA [2].

The annualized rates of all type infections and SBI in our analysis were consistent with findings from other studies on weekly Hizentra^®^ administration [9], which reported 3.10 and 0.03 per patient-years, respectively.

In this pooled analysis, the subject who had a SBI was an overweight fifteen-year-old female patient who was treated with biweekly Hizentra^®^ (IBIS) and was affected by CVID but had no other preexisting pathologies. At the 6-month visit, she was diagnosed with a severe bacterial infection, and she received antibiotic therapy for 17 days. Even though the patient was slightly underdosed (monthly IgG dose = 16.23 g, 103 mg/kg per biweekly dose), she maintained a trough serum IgG level higher than 5 g/L and remained free from infections until this event. This SBI was an exception in her clinical history.

The proportion of pediatric patients treated with antibiotics after an infection (SBI or non-SBI) in the IBIS study was similar to that in the whole study population (61.5% vs. 62.9%, respectively) [18, 19]. The mean time of antibiotic exposure of pediatric patients during the prospective phase of IBIS was 18.9 days, while the median time of antibiotic exposure in the whole population of the same study was 7 days. Therefore, children affected by PID are at a higher risk of infection than adults affected by PID.

A study based on patients’ antibiotic sensitivity profiles found that antibiotic resistance was higher among PID patients than among immunocompetent patients [23]. Although data in the literature are scarce, it is also possible to speculate that IgG replacement therapy may be helpful for combating antibiotic resistance. In vitro and in vivo studies showed that IgG preparations may increase the killing activity of neutrophils against multidrug-resistant bacteria [24–26]. According to a clinical study, IgG therapy may be helpful for treating infections due to multidrug-resistant bacteria in non-PID patients [27]. One case report on a CVID patient found that IgG therapy was effective against multidrug-resistant bacteria [28].

Data from these 2 observational studies demonstrate that weekly and biweekly administrations of Hizentra^®^ to PID children are similarly effective, thus supporting the different dosing regimens allowed by SCIG. Indeed, over time, the European Medical Agency (EMA) has updated the guidelines for 20%-concentrated SCIG administration according to data from pharmacometric simulations and real-world evidence, allowing further usage flexibility. Currently, 20%-concentrated SCIG formulations can be administered at higher volumes and speeds per infusion site if tolerated by the patient or more frequently in small volumes with the manual push technique (MPT) [29], thus allowing the regimen to be tailored to the patient’s needs and preferences.

This analysis has several limitations. As in any rare disease, the sample size was relatively small, however the statistical analysis of our 28 patients could detect as significant a difference of about 1.3 g/L in the serum IgG trough levels. Needless to say, from a clinical point of view, such difference is per se not clinically significant since in the previous studies the mean serum IgG trough levels had a protective range between 7 and 10 g/L [18, 20]. Furthermore, as a post hoc analysis, data were not collected ad hoc, thus preventing the evaluation of relevant clinical issues such as prophylactic antibiotic use. Also, data from the SHIFT study may have been affected by seasonal bias since the observation period was 6 months, and in many patients, follow-up started during spring, thus eluding the months at higher risk of infections. This could explain the different types of infections observed between the two groups (Table 3); specifically, patients in the SHIFT study did not experience any laryngitis, otitis, or sinusitis (Fig. 4).

In conclusion, to our knowledge, this is the first study that indirectly compares weekly and biweekly SCIG regimens in PID populations through post hoc analyses, which were described prospectively. Weekly and biweekly administrations of Hizentra^®^ appear similarly effective in PID pediatric patients in normal clinical practice.

## Data Availability

CSL will consider requests to share individual patient data (IPD) that are received from only systematic review groups or bonafide researchers. CSL will not process or act on IPD requests until 12 months after article publication on a public website. An IPD request will not be considered by CSL unless the proposed research question seeks to answer a significant and unknown medical science or patient care question. Applicable country-specific privacy and other laws and regulations will be considered and may prevent sharing of IPD. Requests for use of IPD will be reviewed by an internal CSL review committee. If the request is approved and the researcher agrees to the applicable terms and conditions in a data sharing agreement, an IPD that has been appropriately anonymized will be made available. Supporting documents, including the study protocol and Statistical Analysis Plan, will also be provided. For information on the process and requirements for submitting a voluntary data sharing request for IPD, please contact CSL at clinicaltrials@cslbehring.com.

## Abbreviations

APDS: Activated PI3K-delta syndrome
ARA: Autosomal recessive agammaglobulinemia
BMI: Body mass index
CI: Confidence interval
CVID: Common variable immunodeficiency
Δ: Absolute difference
DGS: DiGeorge syndrome
FDA: Food and Drug Administration
IgGSD: IgG subclass deficiency
IGRT: Immunoglobulin replacement therapy
IRR: Incidence rate ratio
IVIG: Intravenous immunoglobulin
PAD: Primary antibody deficiency
PID: Primary immunodeficiency
PK: Pharmacokinetic model
Q1W: Weekly posology
Q2W: Biweekly posology
RR: Risk ratio
SBI: Serious bacterial infections
SCID: Severe combined immunodeficiency
SCIG: Subcutaneous immunoglobulin
SD: Standard deviation
UnPAD: Unclassified primary antibody deficiency
XLA: X-linked agammaglobulinemia

## References

[1] Berger M (2008). Principles of and advances in immunoglobulin replacement therapy for primary immunodeficiency. Immunol Allergy Clin North Am..

[2] Food and Drug Administration (FDA). Safety, efficacy, and pharmacokinetic studies to support marketing of immune globulin intravenous (human) as replacement therapy for primary humoral immunodeficiency. Guidance for industry. 2008 https://www.fda.gov/media/124333/download.

[3] Stiehm ER (1997). Human intravenous immunoglobulin in primary and secondary antibody deficiencies. Pediatr Infect Dis J..

[4] Bonagura VR, Marchlewski R, Cox A, Rosenthal DW (2008). Biologic IgG level in primary immunodeficiency disease: the IgG level that protects against recurrent infection. J Allergy Clin Immunol..

[5] Chapel HM, Spickett GP, Ericson D, Engl W, Eibl MM, Bjorkander J (2000). The comparison of the efficacy and safety of intravenous versus subcutaneous immunoglobulin replacement therapy. J Clin Immunol.

[6] Wasserman RL, Melamed I, Nelson RP, Knutsen AP, Fasano MB, Stein MR (2011). Pharmacokinetics of subcutaneous IgPro20 in patients with primary immunodeficiency. Clin Pharmacokinet.

[7] Rojavin MA, Hubsch A, Lawo JP (2016). Quantitative evidence of wear-off effect at the end of the intravenous IgG (IVIG) dosing cycle in primary immunodeficiency. J Clin Immunol.

[8] Högy B, Keinecke HO, Borte M (2005). Pharmacoeconomic evaluation of immunoglobulin treatment in patients with antibody deficiencies from the perspective of the German statutory health insurance. Eur J Health Econ..

[9] Jolles S, Rojavin MA, Lawo JP, Nelson R, Wasserman RL, Borte M (2018). Long-term efficacy and safety of Hizentra^®^ in patients with primary immunodeficiency in Japan, Europe, and the United States: a review of 7 Phase 3 Trials. J Clin Immunol.

[10] Sultan S, Rondeau É, Levasseur MC, Dicaire R, Decaluwe H, Haddad É (2017). Quality of life, treatment beliefs, and treatment satisfaction in children treated for primary immunodeficiency with SCIg. J Clin Immunol.

[11] Gardulf A, Nicolay U, Math D, Asensio O, Bernatowska E, Böck A (2004). Children and adults with primary antibody deficiencies gain quality of life by subcutaneous IgG self-infusions at home. J Allergy Clin Immunol..

[12] Hagan JB, Fasano MB, Spector S, Wasserman RL, Melamed I, Rojavin MA (2010). Efficacy and safety of a new 20% immunoglobulin preparation for subcutaneous administration, IgPro20, in patients with primary immunodeficiency. J Clin Immunol.

[13] Jolles S, Bernatowska E, de Gracia J, Borte M, Cristea V, Peter HH (2011). Efficacy and safety of Hizentra^®^ in patients with primary immunodeficiency after a dose-equivalent switch from intravenous or subcutaneous replacement therapy. Clin Immunol..

[14] Kanegane H, Imai K, Yamada M, Takada H, Ariga T, Bexon M (2014). Efficacy and safety of IgPro20, a subcutaneous immunoglobulin, in Japanese patients with primary immunodeficiency diseases. J Clin Immunol.

[15] Landersdorfer CB, Bexon M, Edelman J, Rojavin M, Kirkpatrick CM, Lu J (2013). Pharmacokinetic modeling and simulation of biweekly subcutaneous immunoglobulin dosing in primary immunodeficiency. Postgrad Med.

[16] Sidhu J, Rojavin M, Pfister M, Edelman J (2014). Enhancing patient flexibility of subcutaneous immunoglobulin G dosing: pharmacokinetic outcomes of various maintenance and loading regimens in the treatment of primary immunodeficiency. Biol Ther..

[17] Wasserman RL, Stein MR, Younger ME, Fatteh S, Haddad E (2016). 20% subcutaneous immunoglobulin dosed biweekly for primary immunodeficiency. Ann Allergy Asthma Immunol.

[18] Vultaggio A, Azzari C, Ricci S, Martire B, Palladino V, Gallo V (2018). Biweekly Hizentra^®^ in primary immunodeficiency: a multicenter, Observational Cohort Study (IBIS). J Clin Immunol.

[19] Canessa C, Gallo V, Pignata C, Trizzino A, Graziani S, Martire B (2019). Subcutaneous immunoglobulin twenty percent every two weeks in pediatric patients with primary immunodeficiencies: subcohort analysis of the IBIS study. Pediatric Allergy Immunol Pulmonol..

[20] Canessa C, Iacopelli J, Pecoraro A, Spadaro G, Matucci A, Milito C (2017). Shift from intravenous or 16% subcutaneous replacement therapy to 20% subcutaneous immunoglobulin in patients with primary antibody deficiencies. Int J Immunopathol Pharmacol..

[21] Cacciari E, Milani S, Balsamo A, Spada E, Bona G, Cavallo L (2006). Italian cross-sectional growth charts for height, weight and BMI (2 to 20 yr). J Endocrinol Invest.

[22] Pecoraro A, Ricci S, Vultaggio A, Boggia GM, Spadaro G, SHIFT and IBIS Study Groups (2020). Correlations among subcutaneous immunoglobulin dosage, immunoglobulin G serum pre-infusional levels and body mass index in primary antibody deficiency patients: a pooled analysis from the SHIFT/IBIS Studies. Clin Drug Investig..

[23] Mohammadinejad P, Ataeinia B, Kaynejad K, Zeinoddini A, Sadeghi B, Hosseini M (2015). Antibiotic resistance in patients with primary immunodeficiency disorders versus immunocompetent patients. Expert Rev Clin Immunol..

[24] Farag N, Mahran L, Abou-Aisha K, El-Azizi M (2013). Assessment of the efficacy of polyclonal intravenous immunoglobulin G (IVIG) against the infectivity of clinical isolates of methicillin-resistant Staphylococcus aureus (MRSA) in vitro and in vivo. Eur J Clin Microbiol Infect Dis.

[25] Matsuo H, Itoh H, Kitamura N, Kamikubo Y, Higuchi T, Shiga S (2015). Intravenous immunoglobulin enhances the killing activity and autophagy of neutrophils isolated from immunocompromised patients against multidrug-resistant bacteria. Biochem Biophys Res Commun.

[26] Itoh H, Matsuo H, Kitamura N, Yamamoto S, Higuchi T, Takematsu H (2015). Enhancement of neutrophil autophagy by an IVIG preparation against multidrug-resistant bacteria as well as drug-sensitive strains. J Leukoc Biol.

[27] Shan LS, Liu X, Kang XY, Wang F, Han XH, Shang YX (2017). Effects of methylprednisolone or immunoglobulin when added to standard treatment with intravenous azithromycin for refractory *Mycoplasma pneumoniae* pneumonia in children. World J Pediatr..

[28] Chao J, Yumei Z, Zhiqun W, Yang Z, Xuguang S (2013). Multidrug-resistant bacteria induce recurrent keratoconjunctivitis in a patient with common variable immunodeficiency: case report and literature review. Cornea.

[29] Shapiro RS (2013). Subcutaneous immunoglobulin: rapid push vs infusion pump in pediatrics. Pediatr Allergy Immunol.

